# An Unusual Case of Lethal *Strongyloides stercoralis*-Associated Chronic Fulminant Colitis in an Immunocompetent Patient

**DOI:** 10.1155/2024/4223529

**Published:** 2024-06-27

**Authors:** Joseph Do Woong Choi, Rukmini Kulkarni, Aswin Shanmugalingam, Charlotte Kwik, Indy Sandaradura, Jasveen Renthawa, Matthew R. Watts, James Wei Tatt Toh

**Affiliations:** ^1^ Department of Colorectal Surgery Westmead Hospital, Corner Hawkesbury Road and Darcy Roads, Westmead, New South Wales, Australia; ^2^ Faculty of Medicine and Health The University of Sydney, Sydney, NSW, Australia; ^3^ Centre for Infectious Diseases and Microbiology Institute of Clinical Pathology and Medical Research New South Wales Health Pathology and Sydney Institute for Infectious Diseases Westmead Hospital University of Sydney, Sydney, New South Wales, Australia; ^4^ Department of Tissue Pathology an Diagnostic Oncology Institute of Clinical Pathology and Medical Research Westmead Hospital University of Sydney, Sydney, New South Wales, Australia

## Abstract

A 70-year-old immunocompetent Lebanese male presented with 3-month history of watery diarrhoea and abdominal pain after recently arriving to Australia from Lebanon. He had a colectomy for an iatrogenic bowel perforation associated with a colonoscopy in Lebanon several months prior. His computed tomography (CT) scan demonstrated pancolitis. Stool culture and polymerase chain reaction (PCR) were positive for *Strongyloides stercoralis*. Despite *Strongyloides* treatment and total parenteral nutrition, his pancolitis unexpectedly persisted despite negative stool cultures, and the patient failed to progress over several weeks with worsening abdominal pain. A colectomy was considered. However, due to his recent myocardial infarct requiring cardiac stenting, his anticoagulant and antiplatelets could not be ceased for at least 3 months without significant cardiac risk. After hospitalisation for several weeks in Australia, he was discharged against medical advice and flew back to Lebanon, where he presented with worsening pain and underwent a subtotal colectomy. Unfortunately, he developed multiorgan failure and died 3 weeks following his colectomy. *Strongyloides*-related pancolitis is a rare condition in immunocompetent adults that has the potential to persist and be lethal, despite microbiological antiparasitic eradication.

## 1. Introduction

*Strongyloides stercoralis* is an intestinal nematode estimated to infect over 600 million people worldwide, mostly in tropical and subtropical areas [1]. Parasitic adult females reside in the duodenum or stomach and produce eggs that hatch in the mucosa [2]. After a series of moults, larvae either progress through an autoinfective pathway, where they penetrate the wall of the large intestine or skin to reinfect the host, or larvae are passed into the environment through faeces, where other hosts can be infected [2, 3]. Due to the capacity for autoinfection, strongyloidiasis can persist for decades in people who have migrated from endemic areas.

Chronic infection may be asymptomatic or be associated with dermatological, respiratory, or gastrointestinal symptoms, with colitis in an immunocompetent host being rarely described [2, 4]. In immunocompromised hosts, there is a shift in host-parasite balance, with an accelerated autoinfection, and large numbers of larvae disseminating through the tissues of the body with a high mortality [5]. Here, we present an unusual case of persistent pancolitis in an immunocompetent patient, associated with strongyloidiasis that was unresponsive to antiparasitic therapy, with pancolitis subsequently complicated by perforated diverticulosis.

### 1.1. History

A 70-year-old Middle Eastern male presented to the emergency department with a three-month history of diarrhoea, vomiting, abdominal distention, and generalised abdominal discomfort. This was associated with 17 kilograms of unintentional weight loss. Several months prior to this presentation, while he was in Lebanon, he had an iatrogenic perforation of the sigmoid colon during a colonoscopy for investigation of per-rectal bleeding requiring an emergency laparotomy and a segmental colectomy with covering ileostomy which was reversed three months later. Five months after reversal, he developed diarrhoea, vomiting, and abdominal pain and was readmitted to hospital. During this admission, he developed a myocardial infarction which required a coronary angiogram and placement of a drug-eluting stent. He had a history of atrial fibrillation, hypertension, dyslipidaemia, and gastrointestinal reflux disease. His regular medications included aspirin, clopidogrel, and apixaban. There was no history of corticosteroid usage nor other immunosuppressant agents. He did not have any personal or family history of colorectal cancer nor inflammatory bowel disease (IBD). He was a current smoker with a 20-pack-year history. There was no history of travel to other areas endemic for strongyloidiasis, such as Asia and the Pacific, sub-Saharan Africa, and Latin America/Caribbean, nor contact with individuals with known *Strongyloides* infection. He left Lebanon, as he was concerned he was deteriorating, and flew to Australia, where he presented to the emergency department on arrival.

### 1.2. Physical Examination

His vital signs were normal. The abdomen was distended, soft with mild generalised tenderness without peritonism. There was a palpable mass in the left mid-abdomen but no clinical features of peritonitis. Digital rectal examination was unremarkable. There were no skin rashes. There were no pulmonary infiltrates on the chest X-ray.

### 1.3. Initial Laboratory and Radiological Investigations

**Figure 1:**
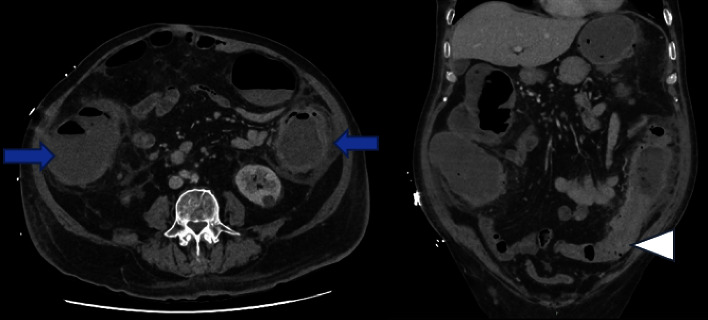
Abdominopelvic CT scans (axial and coronal views) demonstrating pancolitis (blue arrow) with a stricture in the mid-descending colon (white arrowhead) and dilated large bowel loops proximally.

Laboratory findings demonstrated an elevated white cell count (WCC) of 15.2 × 10^9^/L (reference range 3.7–9.5), C-reactive protein (CRP) of 99 mg/L (reference range ≤4), serum creatinine of 101 *μ*mol/L (reference range 60–110), serum potassium of 2.6 mmol/L (reference range 3.2–5.0), and serum albumin of 23 g/L (reference range 35–50). A contrast-enhanced computerized tomography (CT) scan of the abdomen and pelvis demonstrated pancolitis, multiple diverticulosis throughout the length of colon, and radiological features of a large bowel obstruction with a potential stricture in the distal descending colon (Figure 1).

Stool agar plate culture was positive with a moderate level of larvae present and *Strongyloides stercoralis* were identified on microscopy. There was no evidence of *Clostridioides difficile* in the stool. *Strongyloides* species were also confirmed on stool polymerase chain reaction (PCR) (see Appendix 1 for details of primers and probes used) [6], and there was no peripheral blood eosinophilia. Blood cultures were also positive for carbapenemase-producing *Escherichia coli* (CPE) and the presence of a New Delhi metallo-*β*-lactamase (NDM) gene was confirmed on PCR. The infectious diseases team was consulted, and he was initially commenced on two days of ivermectin 18 mg daily orally for *Strongyloides* treatment. For the management of the CPE bacteraemia and colitis, he was commenced on amikacin 1750 mg daily intravenously for 2 weeks, trimethoprim/sulfamethoxazole 160 mg/800 mg twice daily intravenously for 3 weeks, and metronidazole 500 mg twice daily intravenously for 3 weeks. He was unable to tolerate oral intake and commenced on total parenteral nutrition.

### 1.4. Patient Progress after Initial Investigations

Although the patient demonstrated some initial clinical improvement with the above regimen, his clinical progress plateaued with intermittent fevers, a persistent leucocytosis, and a CRP between 80 and 130 mg/L. His electrolytes, renal function, and albumin slowly improved with total parenteral nutrition within 5 weeks. A repeat abdominopelvic CT scan three weeks postadmission demonstrated worsening pancolitis, without features of ischaemia, nor colonic perforation. Ivermectin was restarted one week after discontinuation at 18 mg daily orally, as well as albendazole 400 mg twice daily orally over 2 weeks.

With a failure to progress and worsening colitis on a CT scan, a total abdominal colectomy was considered. However, due to his recent myocardial infarction, the cardiologist advice was that he should not stop his antiplatelet/anticoagulant therapy for at least 3 months. He was referred to the high-risk clinic, and his perioperative mortality was calculated by the perioperative anaesthetist and estimated at 47.6% as per American College of Surgeons Surgical Risk Calculator [7].

**Figure 2:**
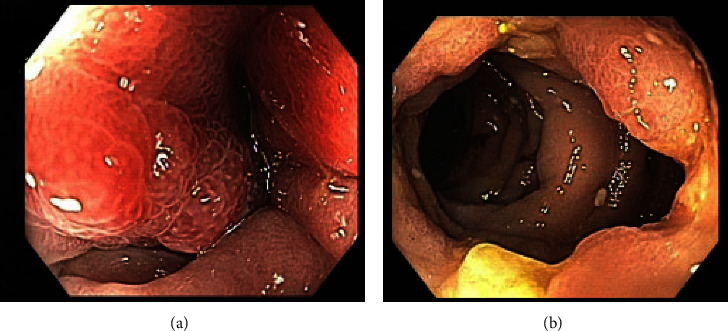
Colonoscopic pictures of an (a) inflammatory stricture, oedema, and (b) punched-out ulcers at the descending colon. Biopsies returned nonspecific reactive changes.

**Figure 3:**
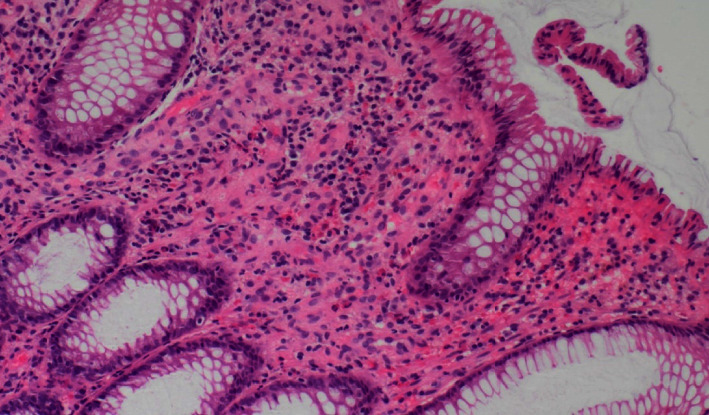
10x hematoxylin and eosin (H&E) stain of the colonic stricture showing mild eosinophilia within the lamina propria.

A colonoscopy demonstrated ulceration and oedema of the left colon with an inflammatory stricture that was able to be traversed (Figure 2). There was sparse mild inflammation in the right colon and diverticular disease. Biopsies of the colonic stricture demonstrated mild eosinophilia within the lamina propria, without granulomas to suggest IBD (Figure 3). Patchy inflammation of the right colon was noted; however, random biopsies of the ascending and transverse colon were unremarkable. The terminal ileum was unremarkable. Biopsies for cytomegalovirus (CMV) were negative on immunohistochemistry.

To assess for other differential diagnoses of fulminant colitis, investigations for ischaemic colitis, autoimmune diseases, vasculitis, CD3 absolute counts, tuberculosis, microsporidia, and hepatitis screens were performed. All these tests were negative. After the antiparasitic therapy, repeat stool culture for *Strongyloides* and PCR were negative.

At the eleventh week of admission, the patient was still unable to tolerate oral intake due to pain, nausea, and vomiting. He was never considered to have immunosuppressive therapy due to concerns of *Strongyloides stercoralis* hyperinfection, and there was no evidence of inflammatory bowel disease. The patient was consented for surgery and explained that he had approximately 40% risk of death. The patient decided to discharge against medical advice. The patient's family was contacted postdischarge against medical advice and we were informed that he had flown back to Lebanon and approximately 1 week after discharge he presented to a hospital in Lebanon with worsening abdominal pains. No endoscopies were performed. A decision was made by the surgeons in Lebanon to perform a subtotal colectomy a few days later.

The histopathology demonstrated a 90 cm segment of large bowel with pancolonic congestive colonic wall, hypertrophy of the muscularis propria, thickened mesocolon, and several diverticulae which were perforated, with several mesocolic abscesses. No *Strongyloides* was seen nor any features of IBD. The patient remained in the intensive care unit postoperatively, developed multiorgan failure, and died approximately 3 weeks after surgery.

A summary of the clinical progress, laboratory findings, and treatments is provided in Table 1.

## 2. Discussion

*S. stercoralis* infection is generally considered in immunosuppressed patients or if there is a history of probable exposure in an endemic area, regardless of elapsed time since exposure [8]. It is symptomatic in 50% of cases, most often manifesting with gastrointestinal symptoms such as nausea, diarrhoea, haematochezia, and diffuse abdominal pains [9]. In immunocompromised patients, it may lead to colitis and hyperinfection syndrome characterised by disseminated *Strongyloides*, with mortality as high as 87% [10].

There have been other rare case reports of chronic colitis in immunocompetent patients associated with *Strongyloides,* but none that persisted after microbiological eradication following antiparasitic activity. Berry et al. reported an immune-competent 16-year-old woman who underwent five colonic operations over five years after a delayed diagnosis of life-threatening chronic haemorrhagic *Strongyloides* colitis [4]. They suggested that the larvae invaded the mucosal walls of the colon at repeated intervals which may be induced by conditions which delay elimination of faecal contents, such as diverticulosis (as was seen in our patient) [4]. Recurrent *S. stercoralis* colitis was reported in a 75-year-old man, 14 months after initial treatment, negative surveillance testing, and subsequent resumption of chemotherapy for his multiple myeloma [11]. In addition, chronic strongyloidiasis may also manifest with paralytic ileus, as reported in a 70-year-old man with 5 months history of recurrent ileus, with resolution of symptoms after ivermectin [12].

In our patient, there was initial strong laboratory evidence of *Strongyloides*-related pancolitis, and the persistence of the colitis after eradication with antiparasitic therapy may be related to the presence of parasite antigen. We propose that a putative mechanism may be related to the persistence of dead parasite antigen without the immune modulatory effects of the live larvae [13]. Serum eosinophilia has not been shown to be an accurate marker for strongyloidiasis, as was the case in our patient, with only 25% of patients with *Strongyloides* infection having raised eosinophil count in a retrospective study [14]. Routine stool examination identifies up to 30% of cases due to low infectious burden and intermittent larval excretion; thus, repeat stool samples are advised to improve the sensitivity of the test [15].

*Strongyloides* colitis on colonoscopy may demonstrate yellowish-white nodules, mucosal erosions, erythema, submucosal haemorrhage, and ulcerations which may alternate between portions of normal mucosa [16, 17]. Our patient had some of these features with appearances that are similar to IBD, including patchy inflammation affecting mostly the left and to a lesser extent, the right colon. Macroscopically, the colitis may be indistinguishable between *S. stercoralis* infection and IBD, leading to misdiagnosis. In our patient, IBD was ruled out from histopathology. This distinction is important, as three case reports misidentified *Strongyloides* colitis by initially treating a patient with immunosuppressive therapy on a presumptive diagnosis of IBD, leading to hyperinfection, severe sepsis, and death [18–20].

Although prohibitive in this patient, an earlier total abdominal colectomy may have avoided his demise. We have illustrated that if persistent *Strongyloides*-related chronic colitis was left untreated without a radical approach, the natural history was lethal from worsening inflammation, colonic perforation, and sepsis.

The limitations of this case report need to be outlined. Firstly, we are assuming that his persistent pancolitis is directly related to his earlier laboratory diagnosis of *Strongyloides* related colitis, perhaps due to persistence of dead parasite antigen in the colon. A complete biochemical, microbiological, radiological, and histopathological analysis had returned negative for infections, inflammatory, autoimmune, or vascular causes for persistent pancolitis. Secondly, although every attempt was made to retrieve his medical records from Lebanon, we were unable to clearly obtain details on what type of colectomy he had and specifically what led to his multiorgan failure and death postoperatively.

## 3. Conclusion

We report a rare case of lethal *Strongyloides stercoralis*-related pancolitis in an immunocompetent male with no significant risk factors. Although there was microbiological evidence of clearance with antiparasitic therapy, there was unexpected persistence of colitis. We propose a putative mechanism for the chronic colitis, related to ongoing colonic inflammation from persistence of dead parasite antigen, without the immune modulatory effects of the live larvae. Although it was prohibitive for this patient, earlier surgery may have prevented death from persistent pancolitis.

## Figures and Tables

**Table 1:** Summary of the clinical, laboratory findings, and treatment.

Week	Results	Intervention/treatment
1	WCC 15.2 × 10^9^/L (reference range 3.7–9.5); CRP 99 mg/L (reference range ≤4) Stool testing: *Clostridium difficile* toxin B DNA negative by PCR *Shigella* DNA negative by PCR *Shiga* toxin DNA negative by PCR *Campylobacter* DNA negative by PCR *Salmonella* DNA negative by PCR *Giardia lamblia* DNA negative by PCR *Entamoeba histolytica* DNA negative by PCR *Cryptosporidium* DNA negative by PCR *Microspiridia* microscopy negative *Strongyloides* culture positive *Strongyloides* DNA detected by PCR Ova, cysts, and parasites: moderate *Strongyloides* larvae Blood cultures: *Escherichia coli*: carbapenemase gene positive (i) Resistant to augmentin, ampicillin, cefiderocol, ceftriaxone, gentamicin, meropenem, and tazocin (ii) Sensitive to colistin and fosfomycin	IV amikacin 1750 mg OD IV metronidazole 500 mg BD

2	WCC 8.3 × 10^9^/L; CRP 75 mg/L Stool testing: *Giardia lamblia* DNA negative by PCR *Entamoeba histolytica* DNA negative by PCR *Cryptosporidium* DNA negative by PCR Ova, cysts, and parasites: moderate *Strongyloides* larvae seen Serum testing *Strongyloides* antibodies 1–2.56 (normal ratio <0.8) Detected CMV IgG detected; CMV IgM not detected (past infection) Blood culture: negative	PO ivermectin 18 mg OD two days of the week IV amikacin 1750 mg OD IV metronidazole 500 mg BD IV trimethoprim/sulfamethoxazole 160 mg/800 mg BD IV

3	WCC 12.6 × 10^9^/L; CRP 76 mg/L Hepatitis B surface antigen not detected Hepatitis B core antibody not detected Hepatitis B surface antibody not detected Stool testing: *Strongyloides* nucleic acid detection positive	IV amikaicin 1750 mg OD IV metronidazole 500 mg BD IV trimethoprim/sulfamethoxazole 160 mg/800 mg BD

4	WCC 11.2 × 10^9^/L; CRP 123 mg/L Stool testing: *Strongyloides* nucleic acid detection positive *Giardia lamblia* DNA negative by PCR *Entamoeba histolytica* DNA negative by PCR *Cryptosporidium* DNA negative by PCR	PO ivermectin 18 mg OD PO albendazole 400 mg BD V metronidazole 500 mg BD IV trimethoprim/sulfamethoxazole 160 mg/800 mg BD
5	WCC 9.0 × 10^9^/L; CRP 55 mg/L Stool testing: *Strongyloides* nucleic acid detection not detected	PO ivermectin 18 mg OD PO albendazole 400 mg BD IV trimethoprim/sulfamethoxazole 160 mg/800 mg BD Colonoscopy

6	WCC 8.8 × 10^9^/L; CRP 37 mg/L	

7	WCC 9.8 × 10^9^/L; CRP 132 mg/L Serum testings: Acid fast bacilli: negative Immunoglobulin G 16.0 g/L (high) Immunoglobulin A 2.37 g/L (normal) Immunoglobulin M 1.84 g/L (normal) C3 complement level 1.2 g/L (normal) C4 complement level 0.37 g/L (normal) Total protein 52 g/L (normal) Albumin EPG 18 g/L (normal) Alpha1 globulin 2.8 g/L (high) Alpha2 globulin 6.9 g/L (normal) Beta globulin 6.2 g/L (normal) Gamma globulin 17.8 g/L (high) Serum immunoelectrophoresis: normal IEPG, no paraproteins Nuclear antibodies screen: not detected Nuclear antibodies cytoplasmic pattern: not detected RNP antibodies: not detected Sm (Smith) antigen antibodies: not detected SSA/Ro 60 antibodies: not detected Ro 52/TRIM 21 antibodies: not detected SSB/La antibodies: not detected Scleroderma 70 antibodies: not detected Jo-1 antibodies: not detected HLA-B27 allele: not detected Stool testing: *Clostridium difficile* toxin B DNA negative by PCR *Shigella* DNA negative by PCR *Shiga* toxin DNA negative by PCR *Campylobacter* DNA negative by PCR *Salmonella* DNA negative by PCR *Giardia lamblia* DNA negative by PCR *Entamoeba histolytica* DNA negative by PCR *Cryptosporidium* DNA negative by PCR *Microspiridia* microscopy negative Ova, cysts, parasites: negative Blood culture: negative	
8	WCC 11.2 × 10^9^ L/; CRP 60 mg/L	

9	WCC 12.6 × 10^9^/L; CRP 194 mg/L	

10	WCC 7.0 × 10^9^/L; CRP 135 mg/L	

11	Discharged against medical advice	

12–15	Presented to a hospital in Lebanon	Subtotal colectomy

WCC, white cell count; CRP, C-reactive protein; IV, intravenous; OD, once a day; BD, twice a day; DNA, deoxyribonucleic acid; PCR, polymerase chain reaction; CMV, cytomegalovirus; EPG, electropherogram; IEPG, immunoelectrophoresis serum globulin; RNP, ribonucleoprotein; SSA, Sjögren's syndrome A; SSB, Sjögren's syndrome B.

## Data Availability

The data that support the findings of this study are available from the corresponding author upon reasonable request.

## Appendix

### A. Primers and Probes Used for *Strongyloides stercoralis* Identification

STR forward primer:

- Stro 185-1530F 5_-GAATTCCAAGTAAACGTAAGTCATTAGC-3_101 bp

STR reverse primer

- Stro185–1630R 5_-TGCCTCTGGATATTGCTCAGTTC-3

STR probe

- Stro185–1586T FAM-5_-ACACACCGGCCGTCGCTGC-3_-BHQ1
